# The potential of the hospital-based Health Technology Assessment: Results of a world-wide survey

**DOI:** 10.1017/S0266462325000108

**Published:** 2025-03-18

**Authors:** Rossella Di Bidino, Iga Lipska, Monika Kukla, Marina von Pinoci, Sara Consilia Papavero, Marco Marchetti, Laura Sampietro-Colom, Americo Cicchetti

**Affiliations:** 1 Graduate School of Health Economics and Management, Università Cattolica del Sacro Cuore (ALTEMS), Rome, Italy; 2 Department of Health Technologies and Innovation, Fondazione Policlinico Universitario Agostino Gemelli IRCCS, Rome, Italy; 3 Hospital Based-HTA Interest Group, Health Technology Assessment International, Edmonton, AB, Canada; 4 Health Policy Institute, Gdańsk, Poland; 5 Clinical Research Centre, Hôpitaux Universitaires de Genève, Geneve, Switzerland; 6 HTA Unit, Italian National Agency for Regional Healthcare Services (AGENAS), Rome, Italy; 7 Assessment of Innovations and New Technologies Unit, Hospital Clínic, University of Barcelona, Barcelona, Spain; 8 Directorate of Health Planning, Italian Ministry of Health, Rome, Italy

**Keywords:** Health Technology Assessment, hospital, survey, decision-making, HB-HTA

## Abstract

**Objectives:**

Hospital-Based Health Technology Assessment (HB-HTA) is a heterogeneous phenomenon constantly evolving to respond to the needs of decision-makers at the hospital level. In 2023, The HB-HTA Interest Group of Health Technology Assessment International (HTAi) surveyed HB-HTA activities with the aim to provide an updated description of the actual scenario.

**Methods:**

An online survey was conducted to gather data on the main characteristics of hospitals, HB-HTA activities, outputs, role in the decision-making processes, dissemination and training activities, and their interaction and collaboration with other stakeholders and HTA-related regulations. Finally, the survey collected feedback on the perception of and current barriers to HB-HTA. Three categories of responders were identified: Both hospitals performing and not performing HTA and policymakers.

**Results:**

Eighty-seven responses were collected from twenty-eight countries. Nearly half of the responders (*n* = 41) conducted HB-HTA, whereas eighteen consisted of hospitals not performing HTA, and twenty-eight were policy makers. HB-HTA was performed mainly in hospitals with >500 beds. HB-HTA units were organized in 40 percent of cases as an “independent group.” The survey showed that HTA units could contribute to all the steps of the decision-making processes, whereas the impact of the assessments on the decisions was mainly perceived as a medium. Furthermore, HB-HTA was not seen as a duplication of effort, even without specific regulations.

**Conclusions:**

The survey highlighted the role of HB-HTA in hospital decision-making supporting the vision of HB-HTA as one of the actors in the HTA ecosystem, the success of which depends on collaboration with other stakeholders.

## Introduction

Hospital-Based Health Technology Assessment (HB-HTA) comprises the implementation of HTA methods and activities in and for hospitals to respond to specific questions on the introduction and management of health technologies in hospitals. It allows hospitals to become more efficient by optimizing the adoption and use of health technologies and avoiding inappropriate investments (1;2).

In 2007, the Hospital-Based Health Technology Assessment Interest Group (HB-HTA IG) of Health Technology Assessment International (HTAi) conducted an international survey (3) to determine who performed HTA “in” hospitals. It investigated how HTA rationales, methods, and tools were adapted within hospitals and other healthcare organizations to support managerial decision-making or clinical practice. Heterogeneity in HB-HTA processes, goals, and available resources emerged.

From 2012 to 2016, the EU-funded project “Adopting Hospital-Based Health Technology Assessment in the EU” (AdHopHTA) (1;2) aimed to enhance the use and impact of high-quality HTA within hospital settings. One of its key achievements was the development of the Handbook of HB-HTA (4), which remains a primary reference in the field of hospital-based HTA. Additionally, the AdHopHTA project developed and validated a specialized glossary for HB-HTA. A. However, because the conclusion of the project, there has been no comprehensive update on the global landscape for the potential role of HB-HTA.

Now, from literature emerged that experts perceive HB-HTA not only as a heterogenous phenomenon but also as a field constantly undergoing rapid transformation to respond to the needs of decision-makers in hospitals (e.g., clinicians, managers) and external stakeholders, including the wide range of the decision-makers in health care ecosystem. National peculiarities are relevant and have thus also been investigated, paying attention to the different maturity levels of HTA at the national level (5;6;7;8). Nevertheless, some features, barriers, and areas for improvement were perceived as common.

Therefore, in 2023, the HTAi HB-HTA IG decided to launch a worldwide survey to collect data on HB-HTA activities and their perceived role and potential and to identify barriers that HB-HTA encounters.

## Methods

The HB-HTA IG’s mission is to gather professionals involved in the use of HTA logic at the hospital level to support both managerial and clinical decision-making processes. It represents the international forum discussion dedicated to HTA in hospitals. For that reason, a survey was developed starting from the issues that emerged during the annual workshop held by the HB-HTA IG during the 2022 HTAi annual meeting. In addition, the AdHopHTA experience and previous survey conducted by the IG in 2007 (3) were considered.

Three categories of responders were defined as follows:

- HB-HTA Doers: defined as hospitals (or healthcare organizations [HCOs]) performing HTA or university centers or research institutions supporting hospitals in HTA activities
- Hospital HB-HTA not Doers: hospitals not performing HTA activities
- Policymakers: national, regional, or local policy makers – HTA agencies included – involved in HTA activities. This category also includes university centers or research institutions not directly supporting hospitals in HTA activities.

Only one response was accepted per organization. Through the survey, data were collected (where applicable) on the main characteristics of the hospitals, HB-HTA activities (including horizon scanning and priority-setting activities), outputs, role in decision-making processes, and other related aspects of HB-HTA, such as dissemination and training activities. Additionally, the external environment was evaluated in terms of interactions and collaborations with various stakeholders (including patients) as well as its recognition within HTA-related regulations. A final survey section was devoted to “critical thinking” to collect feedback on issues such as the perception of HB-HTA as a duplication of work, its role in supporting cost-containment policy and respecting clinicians’ autonomy, and current barriers to HB-HTA at the hospital level. The survey included both open and closed questions. In some cases, participants were asked to rank options or evaluate them using a Likert scale ranging from 0 to 5, where higher scores indicated greater importance.

The survey considered the AdHopHTA project, in relation to HB-HTA Units’ organizational models, categorized by their level of formalization, integration, centralization of authority, and the impact of assessments. It also examined the steps of the decision-making process, ranging from the preliminary analysis of clinical needs to disinvestment decisions, as well as the various types of HTA outputs provided to hospital decision-makers (Supplementary Material S1).

The estimated time to complete was 30 minutes for HTA Doers and 10–15 minutes for Hospitals HB-HTA not Doers and Policymakers. The survey was made accessible to both members and nonmembers of HTAi via the user-friendly Survey Monkey online interface from 31 March to 25 August 2023.

The initiative was disseminated with the support of HTAi, the International Network of Agencies for Health Technology Assessment (INAHTA), the European Health Management Association (EHMA), and the Health Technology Assessment Division (HTAD) of the International Federation of Medical and Biological Engineering (IFMBE). National HTA associations also contributed to survey distribution, as in the case of the Italian Society of Health Technology Assessment (SIHTA), and the Brazilian Company of Hospital Services and authors’ personal networks.

Provisional results of the survey were presented at several key events in 2023: the HTAi Annual Meeting in Adelaide, Australia, in June; a workshop on hot topics in hospital-based research and health technology assessment organized by the HB-HTA IG in October; and the Annual Meeting of the SIHTA in Italy, also in October. This project received financial support from HTAi within its Interest Groups Funding Call 2023.

The current paper provides a general overview of the survey results to subsequently focus on the hospitals where HB-HTA is conducted. Then, information is provided regarding both the external and internal environments of the hospital that support or hinder HB-HTA. The goal is to provide updated evidence on the current state and potential of HB-HTA globally and identify areas for improvement.

## Results

### Survey responders

**Figure 1.**
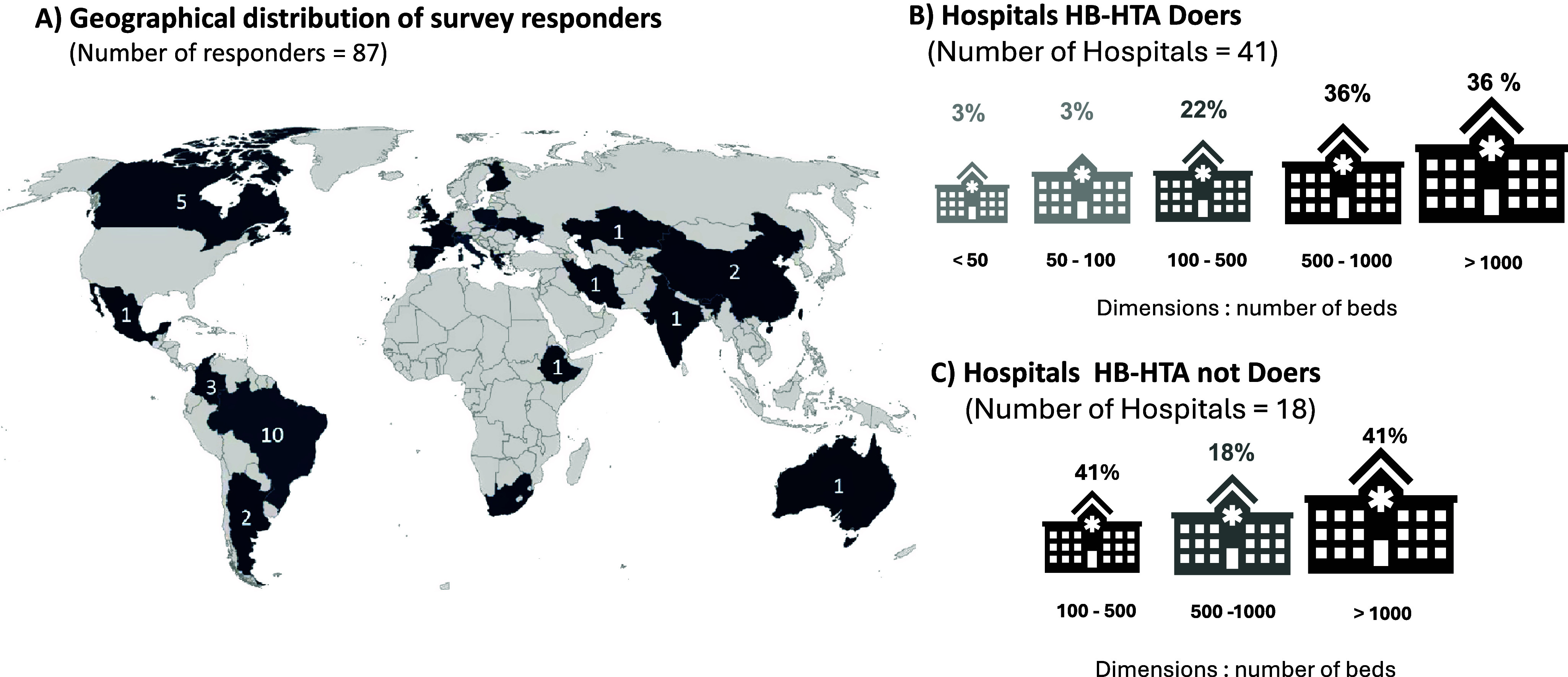
(A) Geographical distribution of survey responders; (B) hospital HB-HTA doers; (C) hospital HB-HTA not doers figure.

Eighty-seven experts from twenty-eight countries responded to the survey. Almost half of the responders (*n* = 41 vs. 33 in the previous survey) conducted HB-HTA, whereas eighteen represented hospitals not currently performing HTA, and twenty-eight were policy makers. Nonmembers of HTAi also responded to the survey (*n* = 44, 51 percent). Interest in the survey also emerged from other stakeholder categories (*n* = 15) that were not included in the study. Because they could not access the full survey, they were excluded from the analysis. As shown in Figure 1A, it was possible to cover all continents even though most responders were from Europe (*n* = 51, 59 percent). Sixteen responses (18 percent) were from South America, eight from Africa, five from Asia, five from North America, and one from Oceania. Supplementary Material S2, Table A1 presents the distribution of responses per country and recipient category.

More than half of the HB-HTA Doers (61 percent) were teaching hospitals, whereas 78 percent of Hospital HB-HTA not Doers were public hospitals/HCOs. In the policy-maker category, 31 percent of responders were from governmental agencies, 31 percent were HTA bodies, and 25 percent were from academia/universities.

### Hospitals performing HB-HTA

**Figure 2.**
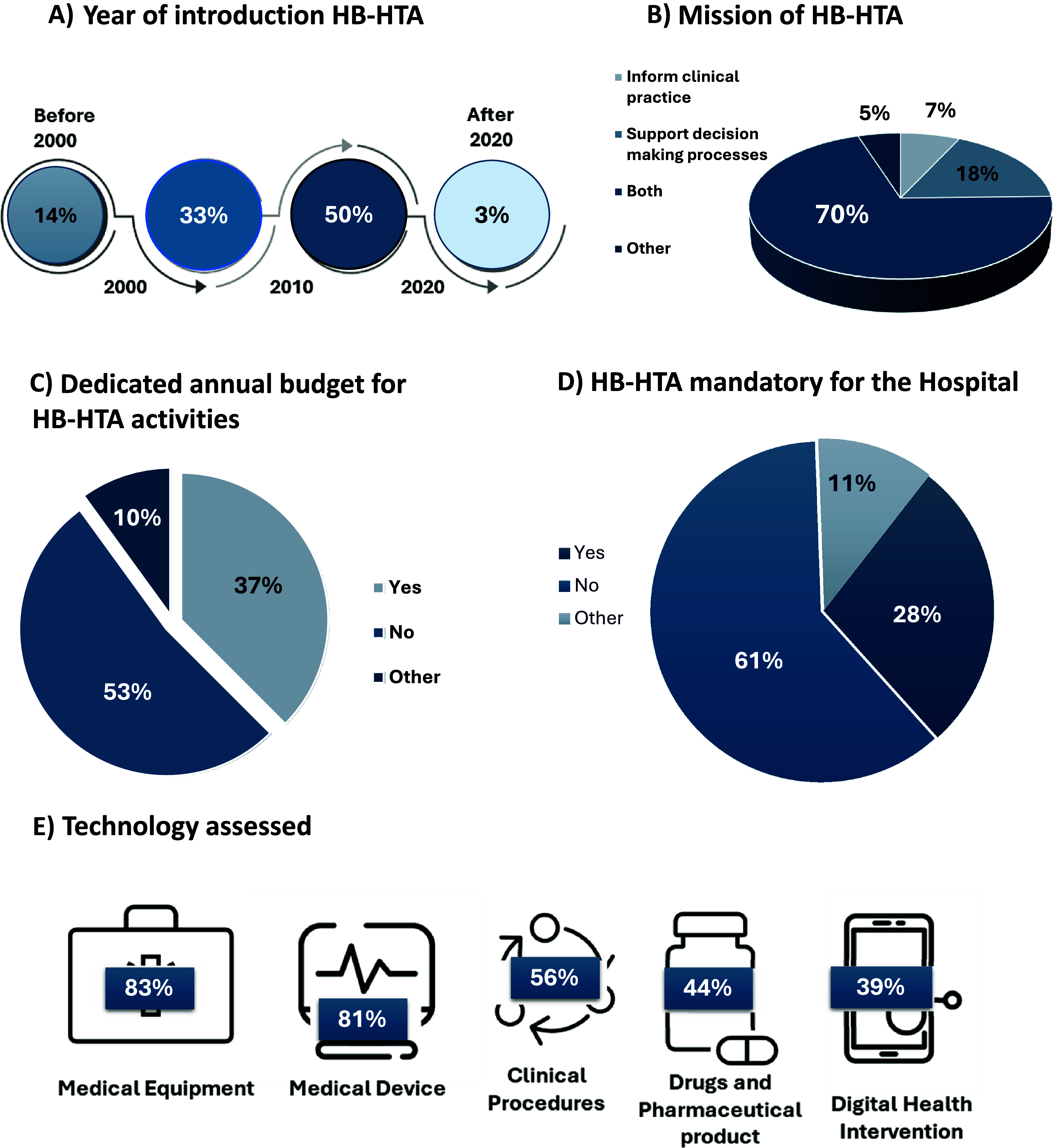
(A) Year of introduction HB-HTA; (B) mission of HB-HTA; (C) dedicated annual budget for HB-HTA activities; (D) HB-HTA mandatory for the hospital; technology assessed.

Hospitals where HTA activities are conducted regularly are the focus of the paper. Our sample showed that HB-HTA was performed mainly in hospitals with more than 500 beds (72 percent; Figure 1B). Thirty six percent of HB-HTA Doers had more than 1000 beds (Figure 1B). Of these hospitals, 50 percent started their HB-HTA activities between 2010 and 2020, whereas 33 percent began between 2000 and 2010. Only one hospital established such activities after 2020 (Figure 2A). The mission of HB-HTA was both to inform clinical practice and to support decision-making processes in 70 percent of cases (Figure 2B).

The organizational model was found to be highly heterogenous: 40 percent of units were integrated-essential HB-HTA units, 25 percent were independent groups, 18 percent were integrated-specialized HB-HTA units, and 10 percent were stand-alone HB-HTA units. In the remaining cases, the model was not fully aligned with the models identified by the AdHopHTA project. The definitions provided to describe the different models are reported in Supplementary Material S1.

Human and financial resources are essential to conduct HTAs and HB-HTAs. In terms of human resources, in decreasing order of frequency, the competencies on which HB-HTA could count included clinicians (78 percent), health economists (63 percent), pharmacists (54 percent), managers (49 percent), public health specialists (46 percent), biomedical engineers (44 percent), and nurses (41 percent). Only 12 percent of the HB-HTA units included a patient representative in their team. Twenty-seven units (66 percent) had full-time permanent staff and 21 (51 percent) had part-time permanent staff. Visiting researcher (24 percent) and internship (29 percent) positions were available but not common. Where available, HB-HTA units typically had a median of three full-time staff members or two part-time staff members. Therefore, despite the attention given to covering different areas of expertise, the dimensions of the HB-HTA units were quite small. In terms of financial resources, only 37 percent of the units had a dedicated budget to conduct their activities (Figure 2C).

### HB-HTA and decision-making processes

**Figure 3.**
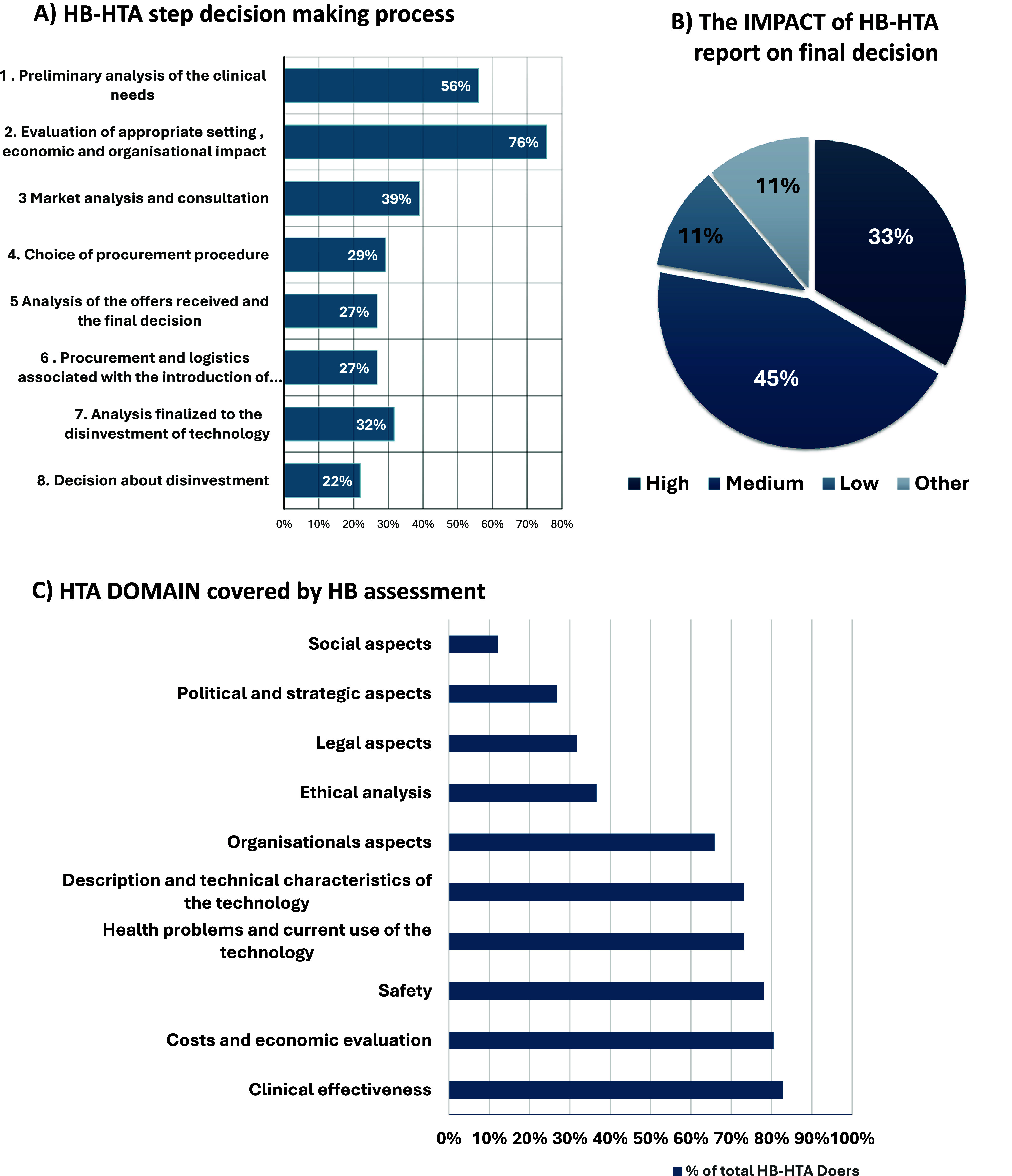
(A) HB-HTA steps decision making process; (B) the impact of HB-HTA on final decision; (C) HTA domain covered by HB assessment.

Regarding the role of HB-HTA in decision-making processes, it was not mandatory in 61 percent of hospitals (Figure 2D). The most common initiator of the process was the heads of the clinical departments (54 percent), and the final decision was mainly in the hands of the chief executive officers (CEOs) (68percent); however, the HTA units participated in all steps of the process, as shown in Figure 3A. The AdHopHTA project outlined eight steps to describe the hospital decision-making process, as detailed in Supplementary Material S1. According to the survey results (Figure 3), the steps to which the HTA units contributed more frequently were the evaluation of the appropriate setting (Step 2, 76 percent of cases), followed by preliminary analysis of clinical needs (Step 1, 56 percent), market analysis (Step 3, 39 percent), and the choice of procurement procedure (Step 4, 29 percent).

HB-HTA was reported directly to the CEO in 68 percent of cases and to the heads of the clinical departments in 51 percent of the responding hospitals.

Responders mainly perceived the impact of the assessments on the final decisions as medium (45 percent) (Figure 3B). A widespread lack of procedures and evidence for regularly assessing the impact of recommendations was identified. When impact assessments were conducted, they were evaluated on a case-by-case basis. Respondents recognized that while final decisions were informed by HTA reports, other factors, such as operational and strategic priorities, were also significant. The extent to which each factor influenced the adoption of health technologies was assessed. The following scenario emerged: economic factors and resources needed were the most influential factors, followed by values (those of patients, clinicians, and hospital managers), presentation and use of evidence (availability, clarity, and strength of empirical scientific evidence on a technology), and organizational factors. The external environment – encompassing factors such as regulatory systems, payment mechanisms, and national or regional regulations – was important, though less influential. The survey focused only on the perception of the impact, not knowing if and how hospitals monitor it. The survey revealed that 59.38 percent of the HB-HTA units had not adopted indicators to evaluate and monitor the impact of their activities.

### HB-HTA activities

Medical equipment and devices were the most commonly assessed health technologies. More than one third (39 percent) of responders assessed digital health interventions (Figure 2E). HB-HTA units were involved in the assessment of COVID-19-related technologies in 47 percent of cases. The majority of hospitals (86 percent) assessed more than one kind of technology.

Focusing on the life cycle of health technologies, very few hospitals dedicated time and resources to horizon scanning (15 percent), whereas 53 percent of units sometimes conducted early assessments. Some responders reported that early HTAs were conducted mainly for technologies related to the hospital’s area of excellence. The same applied to reassessments (47 percent of responders sometimes conducted them, and 9 percent always did). The timing of reassessments ranged from 12 to 36 months after the first report.

On an annual basis, only a few hospitals conducted more than five assessments for a specific type of health technology. Most units produced one – four reports for medical equipment or devices and digital health interventions. Of the responders, 37 percent adopted explicit methods to prioritize their activities, whereas 5 percent proceeded according to the criteria of first-in-first-assessed. The hospital’s strategic plan guided prioritization in 76 percent of cases. Other common criteria adopted included the frequency of the clinical condition (64 percent) and/or healthcare costs (64 percent).

In terms of outputs, mini or rapid HTA reports were more frequently produced (by 73 percent of responders). Half of HB-HTA doers reported also conducting full HB-HTA. The definitions of different kinds of outputs are presented in Supplementary Material S1.

Dissemination of these outputs outside the hospital was not common. Only 29 percent of responders declared sharing them externally. However, the full report was not always shared. Of the HB-HTA units, 62 percent published their findings in scientific journals and 59 percent shared results at conferences/congresses. In terms of transparency of methodologies, only some information on the HTA unit or procedures was shared on the hospital website.

In 79 percent of HB-HTA units, the dedicated staff participated in training activities focused on HTA-related topics, whereas 44 percent of units organized these initiatives by themselves.

### HB-HTA methods

Of the HB-HTA units, 69 percent referred to a specific HTA framework, with a preference for the AdHopHTA (54 percent) and the European Network for Health Technology Assessment – EUnetHTA (25 percent) models. National/agency/hospital-specific frameworks were adopted by 42 percent of responders. Focusing attention on the AdHopHTA framework, all the domains were considered (Figure 3C) some more frequently (as in the case of clinical effectiveness and cost and economic evaluation), others less so (legal aspects). Social aspects were rarely included in HB-HTA, whereas political and strategic factors were investigated only by 27 percent.

To conduct the assessment, scientific literature and data available in the hospital databases (analyzed by 82 percent of hospitals) were considered. The patient perspective was not commonly included in the assessment (31 percent of responders took it into account).

### External environment

A national policy for HTA was common, but a lack of regional policy was evident. Among the respondents, 54 percent (hospitals and policymakers) indicated that only a national policy was in place, whereas 30 percent reported having both national- and regional-level regulations. In 43 percent of cases, no policy – national or regional – explicitly mentioned HB-HTA. Despite this scenario, HB-HTA units commonly collaborated with governmental agencies (in 66 percent of cases), HTA-related network initiatives (66 percent), and academia (61 percent). In some cases, collaborations were conducted also at the international level, mainly within HTA-related networks (32 percent of responders).

HB-HTA units reported the following mainly encountered barriers (in descending order): the role and importance of HB-HTA are not fully perceived, lack of or insufficient budget assigned for HB-HTA activities, and lack of a hospital policy on the integration of HTA into decision-making processes. A lack of human resources and difficulties in finding relevant competencies, despite being real limiting factors, were not reported among the main barriers.

### Perception of HB-HTA

One of the most widespread criticisms of HB-HTA is that it is a potential duplication of work compared to HTAs conducted at the national/regional level. Our survey revealed that most responders (hospitals and policymakers) did not consider it a complete duplication of work. Although only 12 percent of hospitals performing HTA recognized that a partial duplication was possible, 38 percent of hospitals not performing HTA and 27 percent of policy-makers identified that risk.

At the same time, the ability of HB-HTA to support cost-containment policies was recognized by all responders, as was its ability to respect clinicians’ autonomy.

## Discussion

The survey provides an overview of the state of HB-HTA. Despite participation in the survey being voluntary, responses were collected from 28 different countries. Significantly, the survey could retrieve data from forty-one hospitals in sixteen countries where HTA is performed, and not only HTAi members provided responses.

According to the survey, HB-HTA is more likely to be performed in larger hospitals and is not limited to a specific type of technology. HB-HTA units assess mainly medical technologies/devices but also digital health technologies already. In addition, as shown in Figure 2E, all EUnetHTA-AdHopHTA domains were investigated, albeit not with the same frequency. This shows a lack of attention toward Ethical, Legal, and Social Issues (ELSI) domains, whereas organizational aspects play a crucial role in HB-HTA. These results were aligned with a survey conducted in a hospital with 850 beds in 2013 (9), which reported that not only clinical but also organizational factors (such as required investment in infrastructure) were perceived as highly important by most responders. Similarly, in a work by Kildhom (10), a panel of 53 hospital managers from nine European countries reported that clinical, economic, safety, and organizational aspects were the most relevant for decision-making. In addition, the survey confirmed the findings of Ølholm (11) showing that different types of information were not of equal importance to hospital decision-makers and the EUnetHTA’s Core Model was not fully able to respond to the needs of hospital decision-makers.

The role of HB-HTA varies depending on the stage of technology development and its timing relative to the decision-making process. Despite its importance, the allocation of a specific budget for HB-HTA remains rare, which accounts for the limited staff. One critical factor is the assessment of the impact of the HB-HTA activities. Responders perceived a medium impact of HB-HTA on the final decision, given that multiple criteria influenced them. This confirms, as shown by AdHopHTA earlier, that operational and strategic priorities are key from a hospital perspective. The current relevant data show that research regarding HB-HTA impact on decisions is needed, not only a perception of the relevance of HB-HTA to hospital decision-making. Regardless, our survey confirms that the HB-HTA unit contributes to, if not creates, the basis for making informed managerial decisions and improving overall hospital management and evidence based clinical practice, as documented by individual hospital-level experiences (6).

Meanwhile, external to the hospital, a lack of a dedicated legal framework for HB-HTA emerged. The lack of definition of the role, relevance, and area of competence of HB-HTA has a potentially negative influence on its diffusion. Nevertheless, HB-HTA units collaborate with external stakeholders, including national/regional HTA agencies. Being part of HTA networks, also at an internal level, is not rare, demonstrating that HB-HTA is recognized as an actor in the HTA ecosystem despite the absence of specific regulations.

### Strengths

The strengths of the survey are not only providing evidence on the status quo of HB-HTA but also collecting feedback from hospitals not yet performing HB-HTA and policymakers. Their inputs are valuable to better understand the external environment in which HB-HTA operates. The complexity of the survey, represented by its length, allowed it to cover many factors. These included the current workload of HB-HTA units (i.e., the type and number of reports released in a year), the resources available (i.e., financial and human), the role of HTA in different phases of the decision-making process, and the perception (if not yet the measurement) of the impact of assessments.

Compared to previous studies, the survey did not focus only on a specific kind of technology. For instance, in the works of Martelli (12;13), only medical devices were considered. In addition, our survey was not limited to a specific country (7;14;15;16;17;18;19;20;21), region (22), or hospital (14;18;23;24;25;26).

We are aware that an appropriate interpretation of the survey results is possible only by taking into account the results of local studies. However, our survey confirms that some HB-HTA characteristics emerging at the national level are similar across jurisdictions. For instance, according to an online survey conducted in France in 2022 (21), HTA units were more frequent in large hospitals with more than 500 beds. Among our responders, 59 percent of HB-HTA units operated in large hospitals. At the same time, our data showed that some differences exist between countries. In the same French study (21), no hospital reported collaboration with the national HTA agency. Rather, 65.85 percent of the HB-HTA units that responded to our survey declared their collaboration with governmental/national HTA agencies.

In other studies, surveys (17;20;21) or interviews (14;15;16) were commonly used as alternatives to literature reviews. Accordingly, our choice to conduct a survey aligned with established practices, directly engaging individuals in the absence of other easily accessible data sources. As reported, the dissemination of HB-HTA reports is not common.

### Limitations and developments

The survey, as stated above, was voluntary. We managed to reach out to a large panel of experts (*n* = 87) even if they were not equally distributed between countries. Europe was over-represented, along with three countries: two European and one South American (Italy, Brazil, and Poland). Nevertheless, it was possible to collect at least one response per continent. As shown in Supplementary Material S2, for almost all countries on which previous studies were published, our survey was able to capture at least one response, as in the case of China (7), Finland (17;24), Iran (15), and Kazakhstan (25). However, despite evidence of HB-HTA being reported in the literature on Jordan (18), our study could not collect data. In addition, in the case of China, contacting and involving more HB-HTA experts represents a relevant area for improvement in the future. Some studies have been conducted on Low- or Middle-Income Countries (LMICs) or developing countries (19), but these countries are under-represented in our panel of responders. The HB-HTA IG is working on this limitation with targeted initiatives.

We have been aware that this survey is just a first step to guide the future activities of the HB-HTA IG and others. The results revealed that HB-HTA is not an independent activity; however, its initiatives require improved dissemination both within and beyond the hospital. Additionally, the survey highlights common challenges faced by HB-HTA units, consistent with findings from a recent analysis by the HB-HTA IG across seven countries (France, Hungary, Italy, Kazakhstan, Poland, Switzerland, and Ukraine) (27). Both studies underscore that the absence of formal recognition for the role of HB-HTA in national or regional legislation represents a significant external barrier.

Now, the global HB-HTA community could focus its efforts on better defining and defending its role in the national and internal HTA ecosystem, starting from awareness building activities among hospital managers, medical professionals (clinicians, medical bioengineers), and the promotion of dedicated regulations. This is relevant in those countries, such as the EU, where new HTA regulations have been recently launched (Regulation [EU] 2021/2282). Simultaneously, a renewed interest in HB-HTA has emerged.

## Conclusions

The survey conducted by the HB-HTA IG of HTAi provides an updated picture of the role and perception of HB-HTA in 2023. It enriches previous national- or hospital-level analyses and represents a relevant starting point for future studies and initiatives to improve the role of HTA in hospitals and promote HB-HTA. Some aspects of HB-HTA require collaboration with external stakeholders, such as the need for specific regulations, whereas others require internal cooperation (e.g., to promote the role of HTA in decision-making processes among hospital managers, clinicians, and medical bioengineers). Meanwhile, awareness of areas of improvement for HB-HTA is needed. Time and resources should be dedicated to better disseminating and promoting HB-HTA activities. The survey supports the vision of HB-HTA as one of theactors in the HTA ecosystem, the success of which depends on collaboration with other stakeholders.

## Supporting information

Di Bidino et al. supplementary material 1 Di Bidino et al. supplementary material

Di Bidino et al. supplementary material 2 Di Bidino et al. supplementary material

## Supplementary material

The supplementary material for this article can be found at http://doi.org/10.1017/S0266462325000108.
