# Supplementary material for: The potential of the hospital-based Health Technology Assessment: Results of a world-wide survey
Source: Int J Technol Assess Health Care. 2025 Mar 18;41(1):e19. doi: 10.1017/S0266462325000108 (PMC12018856; doi:10.1017/S0266462325000108)
Supplement: Di Bidino et al. supplementary material 1 — Di Bidino et al. supplementary material [file S0266462325000108sup001.docx]

## **Supplementary Material 1**

*Glossary*

| **Organizational models** | |
| --- | --- |
| Independent group | these units operate within the hospital as an “independent group” that provides support for management decisions in a fairly informal way. |
| Integrated-essential HB-HTA unit | these are units of small size, with a limited number of staff members, but who are able to involve many other actors and “allies” in their activities. |
| Stand-alone HB-HTA units | units with usually highly formalised and specialised procedures, acting internally within hospitals and not strongly influenced by the national or regional HTA organisations. |
| Integrated-specialised HB-HTA unit | the functions of the HB-HTA unit are influenced by formal collaboration with the national or regional HTA agency. In general, the involvement of HB-HTA units in the technology adoption process is considered advisable and the HTA-based recommendations are closely followed by hospital decision-makers. |
| **Steps of the decision-making process** | |
| Step 1 | preliminary analysis of the clinical needs (e.g. burden of disease, number of patients that require treatment, available treatment options). |
| Step 2 | evaluation of appropriate setting (level of care) in which the technology is to be used, economic and organisational impact of adopting the technology as well as definition of requisites for tenders. |
| Step 3 | market analysis and consultation. |
| Step 4 | choice of procurement procedure. |
| Step 5 | analysis of the offers received and the final decision. |
| Step 6 | the procurement and logistics associated with the introduction of the technology |
| Step 7 | analysis finalized to the disinvestment of technology |
| Step 8 | decision about disinvestment. |
| **HB-HTA Outputs** | |
| Mini or rapid HTA report | short and structured assessment of the prerequisites for and consequences of using a specific health technology for a specific group of patients at hospital level. |
| Full HB-HTA | a comprehensive, interdisciplinary, systematic assessment of the prerequisites for and consequences of using a specific health technology for a specific group of patients at hospital level. |
| Horizon scan | high-level summary of a new or emerging health technology likely to have a significant impact on the delivery of health care. |
| Reference Lists | bibliographies of existing evidence on a specific topic, arranged by the hierarchy of evidence. |

**Reference**

1. **AdHopHTA Project**. [Internet] The AdHopHTA handbook: a handbook of hospital-based Health Technology Assessment (HB-HTA); c2015. [cited 2023 sept 20] Available from: <https://www.adhophta.eu/handbook>
