# Supplementary material for: The potential of the hospital-based Health Technology Assessment: Results of a world-wide survey
Source: Int J Technol Assess Health Care. 2025 Mar 18;41(1):e19. doi: 10.1017/S0266462325000108 (PMC12018856; doi:10.1017/S0266462325000108)
Supplement: Di Bidino et al. supplementary material 2 — Di Bidino et al. supplementary material [file S0266462325000108sup002.docx]

## **Supplementary Material 2**

*Table  A1. Table: responders by country and category.*

|  | N | | | | percent |
| --- | --- | --- | --- | --- | --- |
| Countries | Hospitals HB-HTA Doers | Hospitals HB-HTA not Doers | Policy Makers | Total |  |
| Albania |  |  | 1 | 1 | 1% |
| Argentina | 1 | 1 |  | 2 | 2% |
| Australia | 1 |  |  | 1 | 1% |
| Belgium |  |  | 2 | 2 | 2% |
| Bhutan |  |  | 1 | 1 | 1% |
| Brazil | 8 |  | 2 | 10 | 11% |
| Canada | 4 |  | 1 | 5 | 6% |
| China | 1 |  | 1 | 2 | 2% |
| Colombia | 2 | 1 |  | 3 | 3% |
| Ethiopia | 1 |  |  | 1 | 1% |
| Finland | 1 |  |  | 1 | 1% |
| France | 2 |  |  | 2 | 2% |
| Greece |  |  | 1 | 1 | 1% |
| India |  |  | 1 | 1 | 1% |
| Iran |  |  | 1 | 1 | 1% |
| Italy | 9 | 5 | 5 | 19 | 22% |
| Kazakhstan | 1 |  |  | 1 | 1% |
| Mexico |  |  | 1 | 1 | 1% |
| Netherlands | 2 |  | 1 | 3 | 3% |
| Poland |  | 9 | 2 | 11 | 13% |
| Scotland |  |  | 1 | 1 | 1% |
| Singapore | 1 |  |  | 1 | 1% |
| Slovakia |  |  | 1 | 1 | 1% |
| South Africa | 2 | 1 | 1 | 4 | 5% |
| Spain | 3 |  | 1 | 4 | 5% |
| Switzerland | 2 | 1 | 1 | 4 | 5% |
| Taiwan |  |  | 2 | 2 | 5% |
| Ukraine |  |  | 1 | 1 | 1% |
| Total | 41 | 18 | 28 | 87 | 100% |
